# Factors Associated With the Presence of Tuberculous Empyema in Children With Pleural Tuberculosis

**DOI:** 10.3389/fped.2021.751386

**Published:** 2021-10-29

**Authors:** Yan-Hua Wu, Jun-Li Wang, Mao-Shui Wang

**Affiliations:** ^1^Department of Lab Medicine, Shandong Provincial Chest Hospital, Cheeloo College of Medicine, Shandong University, Jinan, China; ^2^Department of Lab Medicine, Shandong Public Health Clinical Center, Shandong University, Jinan, China; ^3^Department of Lab Medicine, The Affiliated Hospital of Youjiang Medical University for Nationalities, Baise, China; ^4^Shandong Key Laboratory of Infectious Respiratory Disease, Jinan, China

**Keywords:** tuberculous empyema, children, pleural tuberculosis, risk factor, tuberculosis

## Abstract

**Background:** Until now, the factor of tuberculous empyema (TE) in children with pleural tuberculosis (TB) remains unclear. Therefore, a retrospective study was conducted to assess the factors associated with the presence of TE in children.

**Methods:** Between January 2006 and December 2019, consecutive children patients (≤ 15 years old) with suspected pleural TB were selected for further analysis. Empyema was defined as grossly purulent pleural fluid. The demographic, clinical, laboratory, and radiographic features were collected from the electrical medical records retrospectively. Univariate and multivariate logistic regressions were used to explore the factors associated with the presence of TE in children with pleural TB.

**Results:** A total of 154 children with pleural TB (definite, 123 cases; possible, 31 cases) were included in our study and then were classified as TE (*n* = 27) and Non-TE (*n* = 127) groups. Multivariate analysis revealed that surgical treatment (age- and sex-adjusted OR = 92.0, 95% CI: 11.7, 721.3), cavity (age- and sex-adjusted OR = 39.2, 95% CI: 3.2, 476.3), pleural LDH (>941 U/L, age- and sex-adjusted OR = 14.8, 95% CI: 2.4, 90.4), and temperature (>37.2°C, age- and sex-adjusted OR = 0.08, 95% CI: 0.01, 0.53) were associated with the presence of TE in children with pleural TB.

**Conclusion:** Early detection of the presence of TE in children remains a challenge and several characteristics, such as surgical treatment, lung cavitation, high pleural LDH level, and low temperature, were identified as factors of the presence of TE in children with pleural TB. These findings may improve the management of childhood TE.

## Introduction

According to World Health Organization (WHO) report, in 2018, the global tuberculosis (TB) burden was estimated to be 10.0 million and 1.2 million deaths occurred due to TB (1). Children constitute a significant proportion and account for 11% of total TB cases (1). In addition, at least 200,000 children died from TB in 2018 worldwide. In China, although the incidence of TB in children decreases from 29 per 100,000 in 2008 to 19 per 100,000 in 2017, TB remained the most common bacterial infection in children (2). Due to the considerable TB burden in children, effective measures are important to improve the TB control in children and initiate the diagnosis and treatment of childhood TB at an earlier stage.

Currently, tuberculous empyema (TE) remains a serious threat in China. Our previous study demonstrated that in adults, empyema was reported in 8.9% of patients with pleural TB and significant associations between several clinical characteristics (such as sex, pleural adenosine deaminase (ADA), white blood cell (WBC), and pulmonary TB) and presence of TE were observed (3). In the past decades, the treatment and outcome of empyema in children have been well characterized in several studies. In general, treatments, such as surgical operation or drugs, remain dependent on the empyema stage. For example, the conservative approach remains effective in the management of empyema in children (4); likewise, in adults, surgical management for stage III pediatric empyema is safe, effective, and well tolerated by children (5–7). However, compared with childhood pleural TB without empyema, children TE remains a serious challenge due to the severity, cost, and outcome.

Unfortunately, due to the lack of evidence, the factor of TE in children with pleural TB remains unclear. Therefore, in this retrospective study, we aimed to assess the factors associated with the presence of tuberculous empyema in children with pleural TB. It enables health providers to identify the characteristics of TE in children and develop appropriate strategies to improve the management of childhood TE. Moreover, it would be useful to identify TE from children with pleural TB at a high risk, and then appropriate treatment may be provided timely.

## Materials and Methods

Between January 2006 and December 2019, consecutive children patients (≤ 15 years old) with suspected pleural TB were selected for further analysis. Empyema was defined as grossly purulent pleural fluid (8). Definite pleural TB was defined as positive mycobacterial culture (sputum, pleural effusion, or pleural tissue) or suggested by pathological evidences (such as caseous necrosis, or Langhans' giant cells). Possible pleural TB was diagnosed based on the combinations of clinical symptoms and TB assays (such as TB RT-PCR and acid-fast bacilli (AFB) smear). The demographic (such as age, sex, and weight), clinical (such as symptoms, vital signs, and underling disease), laboratory (such as blood count and chemistry), and radiographic features were collected from the electrical medical records retrospectively.

Statistical analysis was performed using SPSS version 16.0 (SPSS, Chicago, IL, USA). All data were presented as mean ± standard deviation (SD). Univariate logistic regression analysis was performed to assess factors for the presence of TE, and variables with *P-*value <0.1 were included for multivariate logistic regression analysis. Multivariate logistic regression analysis was then performed and the corresponding odds ratios (OR) and 95% confidence interval (CI), adjusted by age and sex, were calculated (9). In addition, to allow a better clinical understanding, continuous variables were transformed into categorical variables according to receiver operating characteristic curve (ROC) analysis. The accuracy of the multivariate model was evaluated using the Hosmer-Lemeshow goodness-of-fit test. The associations between the parameters were estimated using the Spearman correlation test. All tests were 2-sided, and a *P*-value < 0.05 was considered significant.

## Results

### Patient Characteristics

The demographic data, clinical characteristics, laboratory, and radiographic findings collected from enrolled patients were shown in Tables 1, 2. A total of 154 children with pleural TB (definite, 123 cases; possible, 31 cases) were included in our study and then were classified as TE (*n* = 27) and Non-TE (*n* = 127) groups. The patients have a mean age of 12.4 ± 3.3 years, and boys accounted for 64.9% (100 patients). One hundred and three were tested for HIV status, and all were HIV-negative. The weight was measured with a mean of 46.1 ± 16.2 kg. Among the 154 children patients, 93 (60.4%) were from rural areas. The vital signs were as follows: temperature, 37.2 ± 0.9°C; heart rate, 97.6 ± 16.0 beats/min; respiratory rate, 22.5 ± 2.7 breaths/min; blood pressure, 111.4 ± 12.3/69.2 ± 8.5 mmHg.

**Table 1:** Univariate analysis of the demographic data associated with TE in childhood pleural TB.

	**Total (*n*)**	**TE (*n*)**	**Non-TE (*n*)**	***P*-value**	**OR (95% CI)**
*N*	154	27	127		
**Vital signs**					
Temperature (°C)	37.2 ± 0.9	36.8 ± 0.7	37.3 ± 0.9	0.006	0.420 (0.225, 0.781)
Heart rate	97.6 ± 16.0	94.8 ± 16.4	97.3 ± 16.0	0.310	
Respiratory rate	22.5 ± 2.7	22.1 ± 2.5	22.5 ± 2.9	0.325	
Systolic pressure	111.4 ± 12.3	114.2 ± 10.0	111.0 ± 11.8	0.191	
Diastolic pressure	69.2 ± 8.5	70.8 ± 9.1	68.1 ± 7.7	0.275	
**Medical history**					
Contact history of TB	20 (13.0%)	1 (3.7%)	19 (15.0%)	0.147	
Transferred times	2.1 ± 1.0	2.0 ± 1.0	2.2 ± 1.1	0.788	
Transferred from a teaching hospital	91 (59.1%)	16 (59.3%)	75 (59.1%)	0.984	
Times of hospitalization	2.0 ± 1.6	2.9 ± 2.5	1.7 ± 1.3	0.002	1.431 (1.141, 1.793)
Treatment delay (days)	61.8 ± 134.6	60.7 ± 67.6	91.8 ± 177.8	0.965	
Surgical treatment	29 (18.8%)	19 (70.4%)	10 (7.9%)	0.000	27.787 (9.739, 79.288)
**Symptoms and complications**					
Cough	84 (54.5%)	13 (48.1%)	71 (55.9%)	0.463	
Fever (>38°C)	134 (87.0%)	20 (74.1%)	114 (89.8%)	0.034	0.326 (0.116, 0.917)
Chest pain	69 (44.8%)	13 (48.1%)	56 (44.1%)	0.701	
Dyspnea	42 (27.3%)	6 (22.2%)	36 (28.3%)	0.518	
Sputum production	29 (18.8%)	5 (18.5%)	24 (18.9%)	0.964	
Cavity	7 (4.5%)	3 (11.1%)	4 (3.1%)	0.091	
Loculated effusion	27 (17.5%)	10 (37.0%)	17 (13.4%)	0.005	3.806 (1.497, 9.679)
**Clinical chemistry (pleural effusion)**					
Total Protein	48.5 ± 7.2	45.9 ± 8.0	49.4 ± 7.3	0.215	
Total Bilirubin (mmol/L)	8.6 ± 5.6	11.5 ± 10.1	8.2 ± 5.0	0.109	
Adenosine deaminase (U/L)	60.3 ± 28.8	71.5 ± 49.4	61.6 ± 28.4	0.198	
Glucose (mmol/L)	3.3 ± 1.5	2.3 ± 1.7	3.4 ± 1.7	0.029	0.606 (0.386, 0.951)
Lactate dehydrogenase (U/L)	876.7 ± 642.8	1505.4 ± 1308.1	842.1 ± 491.9	0.010	1.001 (1.000, 1.002)
Amylase (U/L)	29.6 ± 10.4	25.7 ± 11.0	32.0 ± 11.6	0.210	
**Other analysis**					
Erythrocyte sedimentation rate (mm/h)	40.8 ± 26.4	29.6 ± 26.0	42.4 ± 27.6	0.021	0.977 (0.958, 0.996)

*TB, tuberculosis; OR, odds ratio; CI, confidence interval*.

**Table 2:** Univariate analysis of the demographic data associated with TE in childhood pleural TB.

	**Total (*n*)**	**TE (*n*)**	**Non-TE (*n*)**	***P*-value**
*N*	154	27	127	
**Demographic characteristics**					
Age (years)	12.4 ± 3.3	12.9 ± 3.0	12.3 ± 3.4	0.366
Sex (male)	100 (64.9%)	17 (63.0%)	83 (65.4%)	0.813
Weight (Kg)	46.1 ± 16.2	46.5 ± 17.0	44.9 ± 14.9	0.889
Rural area	93 (60.4%)	17 (63.0%)	76 (59.8%)	0.763
**Effusion sites**					
Left	62 (40.3%)	10 (37.0%)	52 (40.9%)	0.707
Right	74 (48.1%)	13 (48.1%)	61 (48.0%)	0.991
Both	18 (11.7%)	4 (14.8%)	14 (11.0%)	0.579
**Comorbidity**					
Pulmonary TB	89 (57.8%)	14 (51.9%)	75 (59.1%)	0.492
Tuberculous lymphadenitis	13 (8.4%)	4 (14.8%)	9 (7.1%)	0.200
Milliary TB	7 (4.5%)	2 (7.4%)	5 (3.9%)	0.439
Tuberculous meningitis	4 (2.6%)	1 (3.7%)	3 (2.4%)	0.693
Bronchial tuberculosis	3 (1.9%)	0	3 (2.4%)	0.999
**Clinical Chemistry (serum)**					
Total protein (g/L)	68.7 ± 6.9	68.2 ± 7.2	69.3 ± 7.5	0.673
Albumin (g/L)	38.8 ± 4.8	38.7 ± 5.2	38.7 ± 4.9	0.908
Blood urea nitrogen (mmol/L)	3.9 ± 1.2	3.6 ± 1.0	4.0 ± 1.2	0.174
Creatinine (μmmol/L)	52.3 ± 15.3	55.7 ± 14.7	49.2 ± 13.9	0.227
Glucose (mmol/L)	4.8 ± 1.2	4.8 ± 0.6	4.9 ± 1.6	0.908
Lactate dehydrogenase (U/L)	219.8 ± 71.1	220.6 ± 82.2	208.8 ± 54.0	0.951
**Blood analysis**					
White blood cell (10^9^/L)	7.3 ± 2.6	6.8 ± 2.0	7.4 ± 2.8	0.320
Red blood cell (10^12^/L)	4.4 ± 0.5	4.6 ± 0.5	4.5 ± 0.5	0.114
Hemoglobin (g/L)	121.2 ± 14.2	124.2 ± 18.4	120.4 ± 14.0	0.238
Hematocrit	36.6 ± 3.9	37.3 ± 4.6	36.6 ± 4.0	0.299
Mean corpuscular volume (fL)	82.4 ± 5.1	81.5 ± 5.5	82.3 ± 5.0	0.304
Mean corpuscular hemoglobin (pg)	27.3 ± 2.1	27.1 ± 2.6	27.1 ± 1.8	0.579
Mean corpuscular hemoglobin concentration (g/L)	331.3 ± 12.7	332.2 ± 13.3	329.1 ± 10.3	0.707
Platelet (10^9^/L)	352.8 ± 129.0	333.2 ± 122.8	359.5 ± 146.9	0.393
Neutrophil (10^9^/L)	5.1 ± 6.2	4.2 ± 1.5	4.7 ± 2.3	0.263
Lymphocyte (10^9^/L)	1.7 ± 0.9	1.8 ± 0.7	1.7 ± 0.8	0.832
Monocyte (10^9^/L)	0.8 ± 0.4	0.7 ± 0.3	0.8 ± 0.4	0.307
Coefficient of variation of red cell distribution width (%)	13.9 ± 1.8	14.4 ± 2.1	14.1 ± 2.0	0.129
**Flow cytometry**					
CD19+ (%)	24.8 ± 20.1	19.4 ± 9.5	25.8 ± 19.7	0.432
CD3+ (%)	62.4 ± 13.7	64.4 ± 12.1	61.5 ± 13.5	0.658
CD3+CD4+ (%)	33.1 ± 9.1	35.8 ± 7.8	34.1 ± 9.5	0.379
CD3+CD8+ (%)	24.0 ± 12.0	24.5 ± 10.4	22.4 ± 10.7	0.902
CD3-CD16+CD56+ (%)	11.8 ± 6.2	10.9 ± 5.7	11.6 ± 6.9	0.657
CD4+/CD8+ (%)	2.8 ± 3.9	1.9 ± 1.4	2.8 ± 3.7	0.531

*TB, tuberculosis*.

Twenty (13.0%) patients had a TB contact history, and surgical techniques were employed in the 29 (18.8%) patients. Prior to the admission to our center, most of them (91, 59.1%) were treated at a teaching hospital, and the mean times of hospitalization were 2.0 ± 1.6. The most common symptom was fever (134, 87.0%), followed by cough (84, 54.5%), chest pain (69, 44.8%), dyspnea (42, 27.3%), and sputum production (29, 18.8%). Cavity and loculated effusion revealed by radiographic examinations were observed in 7 (4.5%, 7/154) and 27 (17.5%, 27/154) patients, respectively. In addition, 62 (40.3%, 62/154) patients had effusion on the left side, 74 (48.1%, 74/154) on the right side, and 18 (11.7%, 18/154) on the both-side. Out of the total of 154 study patients, 89 (57.8%) had pulmonary TB, 13 (8.4%) had tuberculous lymphadenitis, 7 (4.5%) had milliary TB, 4 (2.6%) had tuberculous meningitis, 3 (1.9%) had bronchial TB, and the remaining 38 (24.7%) were isolated pleural TB.

Other characteristics, such as clinical chemistry analysis (serum or pleural effusion), blood cell analysis, and flow cytometry analysis, were summarized in Tables 1, 2.

### Univariate and Multivariate Analysis

Table 1 shows the univariate analysis of risk factors, comparing patients with TE with patients without TE. It was found that the presence of empyema was associated with temperature (OR = 0.420, 95% CI: 0.225, 0.781), hospitalization times (OR = 1.431, 95% CI: 1.141, 1.793), surgical treatment (OR = 27.787, 95% CI: 9.739, 79.288), fever (OR = 0.326, 95% CI: 0.116, 0.917), loculated effusion (OR = 3.806, 95% CI: 1.497, 9.679), pleural glucose (OR = 0.606, 95% CI: 0.386, 0.951), pleural LDH (OR = 1.001, 95% CI: 1.000, 1.002), and erythrocyte sedimentation rate (ESR, OR = 0.977, 95% CI: 0.958, 0.996) (all *P* < 0.05).

To make the results as readily understandable as possible, continuous variables were converted into dichotomous categorical variables based on the cut-off points determined with ROC analysis, and the corresponding optimal cut-off values were 37.2°C, 29 mm/h, 2.98 mmol/L, and 941 U/L, for temperature, ESR, pleural glucose, and LDH, respectively. Further multivariate analysis (Hosmer–Lemeshow goodness-of-fit test: χ^2^ = 2.780, d*f* = 8, *P* = 0.947) revealed that surgical treatment (age- and sex-adjusted OR = 92.0, 95% CI: 11.7, 721.3), cavity (age- and sex-adjusted OR = 39.2, 95% CI: 3.2, 476.3), pleural LDH (>941 U/L, age- and sex-adjusted OR = 14.8, 95% CI: 2.4, 90.4), and temperature (>37.2°C, age- and sex-adjusted OR = 0.08, 95% CI: 0.01, 0.53) were associated with the presence of TE in children with pleural TB (Table 3).

**Table 3:** Age- and sex-adjusted OR for risk factors associated with TE in childhood pleural TB.

	**Adjusted (age and sex) OR**	***P*-value**
Surgical treatment	92.0 (11.7, 721.3)	<0.001
Cavity	39.2 (3.2, 476.3)	0.004
Pleural LDH (>941 U/L)	14.8 (2.4, 90.4)	0.004
Temperature (> 37.2°C)	0.08 (0.01, 0.53)	0.009

*TB, tuberculosis; OR, odds ratio; CI, confidence interval*.

## Discussion

Until now, factors that affect the presence of empyema remain uncertain. In this study, the associations between TE and clinical characteristics were assessed in a referral TB hospital. Our study found that surgical treatment, cavity, pleural LDH, and temperature were associated with the presence of TE in children with pleural TB. To our knowledge, this is the first report investigating factors of the presence of TE among children with pleural TB. Our findings may improve the management of childhood TE and help appropriate treatment to be initiated timely.

First, the cavity in the lungs was identified having an association with the presence of empyema. In fact, previously, it is thought that lung cavitation means a high TB burden, high infectivity, and is associated with TE (10). One possible explanation is that cavitation appears to first occur within the parenchymal consolidation and TE may result from an inadvertent rupture into the pleural space (11, 12). This situation is not uncommon (11). In addition, the association suggests an indirect evidence of the mechanism of pleural TB formation that TB strains in lungs disseminate into pleural space directly.

Second, in the study, surgical treatment was also associated with the presence of TE in children. This finding shows that in our study, most of childhood TE patients underwent surgical treatment. To treat the TE and minimize the morbidity and mortality, physicians were required to choose the appropriate procedure. However, the optimal treatment of childhood TE remains unclear (13). In addition, although most of children patients with empyema can be successfully treated with the conservative treatment, the conservative treatment has a disadvantage because of the long duration of hospital stay, especially in cases with advanced stages (4). Hence, surgical treatment, such as decortication, resection, and muscle flap closure, is still necessary in childhood pleural empyema (14). However, the choice of surgical approach may be determined by the stage of TE and the corresponding success rates may be influenced by the stage of the empyema, such as stage I and II (5, 6, 15, 16).

Third, pleural LDH, as a biomarker varied during the progression of pleural inflammation (17), is found to be associated with the presence of TE in children. It is thought that the initial level of pleural LDH reflects the serum level of LDH due to the filtration into the pleural space. In contrast, an increased LDH level is thought to have a cellular origin rather than a filtration origin (18). Similarly, pleural LDH can be used as a biomarker in the diagnosis of empyema. For example, Chen et al. found pleural LDH (≥1000 U/L) to be a maker discriminating complicated parapneumonic pleural effusion and empyema from uncomplicated parapneumonic pleural effusion with an AUC of 0.949 (19). Besides the above mentioned, pleural LDH also was found to be correlated with the duration of fever in patients with empyema, which may reflect the inflammation process (20, 21).

Low temperature was considered as another risk factor of the presence of TE among children with pleural TB. As known, the rise in the temperature due to empyema is a favorable symptom which may aid to shorten the delay in the treatment of TE. Therefore, the progression of developing TE was then stopped or delayed. Similarly, previous studies showed that absence of fever was significantly associated with total delay in patients with TB disease (9, 22–25). Interestingly, in the study, temperature was included in the final model. However, fever was not included in it. This may be explained by that fever, as an initiation symptom, was recalled by the patient before the admission to the hospital and the temperature was measured on the admission. Therefore, disease progression and previous treatment may made a significant impact and lead to the different analysis result. In contrast, a history of prolonged fever was confirmed as a significant clinical predictor for empyema in children (26, 27). Moreover, a longer duration of fever was associated with complicated community-acquired pneumonia, another serious complication as empyema (27). Based on these mentioned, further studies are required to investigate the association between temperature (or fever) and empyema, especially the corresponding biological plausible mechanism.

Although this study gives an insight into the management of childhood TE, the results of this study should be interpreted with some caution. First, retrospective collection of data and smaller number of cases are a significant concern for our study. Second, the study was based on a single center experience and our findings may generalize to other children populations. Furthermore, as the study had a relatively small sample size, a large study of childhood TE may be required to confirm these results.

## Conclusions

Our findings suggest that early detection of the presence of TE in children remains a challenge in practice. Several characteristics, such as surgical treatment, lung cavitation, high pleural LDH level, and low temperature, have been identified as risk factors for the presence of TE in children with pleural TB. These findings may help to improve the diagnosis of TE and initiate appropriate treatment earlier. Moreover, it would also aid in shaping strategies for preventive management of childhood TE.

## Data Availability Statement

The original contributions presented in the study are included in the article/supplementary material, further inquiries can be directed to the corresponding author/s.

## Ethics Statement

This study was conducted at the Shandong Provincial Chest Hospital and confronted for the Helsinki Declaration. The study protocol was approved by the Ethics Committee of Shandong Provincial Chest Hospital. Due to the retrospective nature of this investigation and the anonymous nature of the data collection, this retrospective study was exempt from the need for written informed consent by the Ethics Committee of Shandong Provincial Chest Hospital.

## Author Contributions

M-SW and J-LW: designed the study and supervised data collection. Y-HW: performed statistical analysis and drafted the initial manuscript. M-SW: collected data. All authors approved the final version of the report.

## Funding

This work was supported by the Science Research and Technology Development Plan of Baise City (20203405).

## Conflict of Interest

The authors declare that the research was conducted in the absence of any commercial or financial relationships that could be construed as a potential conflict of interest.

## Publisher's Note

All claims expressed in this article are solely those of the authors and do not necessarily represent those of their affiliated organizations, or those of the publisher, the editors and the reviewers. Any product that may be evaluated in this article, or claim that may be made by its manufacturer, is not guaranteed or endorsed by the publisher.
